# A sympathetic nervous system evaluation of obesity stigma

**DOI:** 10.1371/journal.pone.0185703

**Published:** 2017-10-30

**Authors:** Michael D. Oliver, Subimal Datta, Debora R. Baldwin

**Affiliations:** 1 Department of Psychology, College of Arts and Sciences, The University of Tennessee, Knoxville, Tennessee, United States of America; 2 Department of Anesthesiology, Graduate School of Medicine, The University of Tennessee, Knoxville, Tennessee, United States of America; Technion Israel Institute of Technology, ISRAEL

## Abstract

The portrayal of obesity in the media is often one of negativity. Consequently, it may generate an increase in stigma. Obesity stigma, a form of social discrimination, is responsible for many of the negative psychological and physiological effects on individual wellness. These effects not only impact individual health, but also affect the economy, and ultimately, societal wellness. In an attempt to examine the influence of the media on obesity stigma, this study tested the hypothesis that positive priming would lead to a reduction in obesity stigma. To further our understanding of this relationship, we: 1) examined the role of priming on physiological measures (e.g. salivary alpha amylase and skin conductance) in 70 college students by introducing positive and negative media images of individuals with obesity, and 2) assessed psychological measures (e.g. perceived stress, need to belong, and self-esteem, and Body Mass Index). After the priming manipulation, participants read a vignette depicting the discrimination of an individual with obesity and answered subsequent questions assessing participants’ attributional blame of obesity. Results of this study revealed that priming affects physiological responding to obesity stigmatization. In conclusion, these findings suggest that incorporating positive media images of individuals with obesity may be an effective tool for reducing stigma and the various physiological consequences associated with it, which in turn, can enhance societal health and wellness.

## Introduction

One-fifth of children and adolescents and more than one-third of adults in the United States are obese [1]. In addition to the well-known negative health consequences of obesity, millions of US citizens classified as obese also suffer from ubiquitous discrimination. Obesity stigma, a form of social discrimination, is responsible for a host of negative psychological [2,3,4,5] and physiological [6,7] effects on individual well-being. For example, increased risk of depression [8], decreased self-esteem [9], suicidality [10], elevated blood pressure [11], and dysregulations of stress response systems in the body [12] are some of the detrimental effects various forms of social discrimination places on individual well-being. The media’s negative portrayals of obesity often lead to deleterious consequences not only for the stigmatized target individuals, but also for others who are exposed [13]. These frequent negative media portrayals promote the belief that individuals with obesity lack self-discipline [14]. In addition, higher exposure to mass media outlets is positively correlated with stigmatizing attitudes towards obese children [15]. More importantly, research indicates that the most significant predictor of stigmatizing attitudes towards this group is personal causal attributions [14], and there are significantly more media references alluding to personal responsibility rather than social determinants or attributions of responsibility [16].

The Autonomic Nervous System (ANS) is influenced by psychosocial factors that affect health and disease outcomes [17]. With regard to obesity stigma, the psychophysiological correlates are not well defined. Various studies exploring obesity stigma have almost exclusively examined the stress hormone cortisol, an indicator of long-term stress regulated by Hypothalamic-pituitary-adrenal (HPA) axis activity in the Sympathetic Nervous System (SNS) [18,19,20,21]. In a study by Schvey, Puhl, & Brownell [22] it was reported that exposure to obesity stigma is associated with greater cortisol reactivity in both overweight, as well as, lean female participants. This study posits that although one might not expect mere exposure to stigmatizing material that is not personally relevant to affect them, it could in fact be potentially harmful physiologically. In addition, studies exploring obesity stigma are conducted almost exclusively on women, and the data are limited in terms of men and their responses [23]. In one study, Hebl & Turchin [24] examined obesity stigma in a population of males. In this study, a questionnaire was employed assessing the degree to which men stigmatize obesity. The authors concluded that men both stigmatize obesity, and are stigmatized for being overweight. These aforementioned studies support the premise that obese men and women both experience stigma as a consequence of their weight. Although this study provides data on self-report responses to obesity stigma among men, this particular study did not employ the use of physiological measures, thus research is lacking in terms of how men respond physiologically to this phenomenon in conjunction with self-report.

Although cortisol has been profoundly used as a biomarker in examining stress [25,26], salivary Alpha-Amylase (sAA) has received more modern attention in terms of examining psychosocial stress. In a study by Rohleder et al. [27], it was proposed that protein enzyme alpha-amylase, regulated by the Sympathomedullary Pathway (SAM), was activated in a psychosocial stress-induced paradigm. The SAM system, reactive in response to short term stress, introduced the notion of sAA as a biomarker for psychosocial stress research. Further studies such as Nater et al. [28], van Stegeren, Wolf, & Kindt [29], Nater & Rohleder [30], and Engert et al. [31] have since corroborated the assumption and validated sAA as a non-invasive biomarker of SNS activity for short term evaluations of psychosocial stress. By incorporating sAA, paired with Skin Conductance (SC), a SNS measure of arousal, we believe we can garner a more complete physiological understanding of the phenomenon of stigma as it relates to the sympathetic division of the ANS response to stress. We know that obesity stigma is a psychosocial stressor, however, the research is limited with regard to the autonomic responses as a result of exposure to such stigma.

The purpose of this study was to examine the psychophysiological aspects of obesity stigma. Through priming, an approach designed to influence attitudes and perceptions both implicitly and explicitly, we assessed whether one’s attributional blame for obesity can be modified. Images of individuals with obesity were introduced in both stigmatizing and non-stigmatizing fashion in an attempt to prime participants to think either positively or negatively about obesity. We expected to reduce the negative connotations associated with personal responsibility for obesity in the positive primed group. That is, by altering the associative structure between obesity and negativity, and associating obesity with more positivity, we hypothesize that individuals will subsequently feel less discriminatory and hold less stigmatizing views toward this population. Studies such as Olson & Fazio [32] show that by reassigning evaluative conditioning of an attitude, subsequent attitude change can result. First, we hypothesize that the group exposed to positive non-stigmatizing images of individuals with obesity will exhibit less physiological responsiveness of the SNS than individuals exposed to negative stigmatizing images of individuals with obesity. Moreover, when given an actual discrimination vignette, the positive primed group will place less attributional blame on the obese individual than their negative group counterparts. We anticipate that the positive images will be a less stressful and arousing stimulus because they avoid triggering the societal stigma associated with obesity. We believe that by reducing the negative stigma surrounding obesity, this group’s image in society can be improved.

Using biomarkers of SNS activation, our study is the first to our knowledge, to test the physiological effects of priming on individuals exposed to this form of stigma. To our knowledge, our study is the first to examine if men also respond in similar fashion as women to stigmatizing obesity stimuli from a physiological standpoint. Secondly, we hypothesize that women will be more reactive both psychologically, as well as physiologically, to obesity stigma compared to men. In this study, we aim to expand upon extant findings and provide a more complete picture of the sex responses to obesity stigma by examining whether or not physiological responses to obesity stigma manifest themselves similarly in men and women. Both salivary Alpha-Amylase (sAA) and skin conductance (SC) were employed to measure SNS arousal. Our third hypothesis is that individuals with obesity will have a different perspective in terms of psychological responding than their normal weight counterparts [22]. With regards to need to belong, we expect individuals with obesity to self-report greater need to belong. A number of studies have suggested that overweight individuals may feel ostracized and excluded from normal weight group activities [33,34,35]. Stigma is a form of a social stressor, so we expect higher levels of perceived stress as well as noted by Zeiders, Hoyt, & Adams [12]. Moreover, self-esteem has a social component. According to Myers & Rosen [36], we expect individuals with obesity to report lower self-esteem compared to their normal weight counterparts. Finally, fat phobia is another measure of stigma towards weight. We expect obese participants to have less fat phobic attitudes and place less attributional blame on their in-group compared to the normal weight (out-group) participants.

The current study examines the role of positive and negative media images of individuals with obesity on attitudes and physiological responding in relation to an actual discrimination incident. Our fourth and final hypothesis is that the positive priming manipulation will result in a decrease of stigmatizing/discriminatory views of obesity. More specifically, the positively primed group is expected to exhibit lower levels of sAA and SC post-vignette compared to their negative group counterparts. By positively priming individuals, the feelings of negativity toward the obese individual should be reduced, thus resulting in less stress and arousal from the vignette. Finally, we expect that females will exhibit larger increases in sAA and SC arousal in comparison to males across priming conditions.

## Materials and methods

### Participants

Participants consisted of a total of 70 students ranging from 18–53 years of age (50 female, 20 male; mean age = 20.94, *SD* = 6.11) recruited from undergraduate Psychology courses at a large southeastern university. The participants were primarily White (75.7%). Prior to participation, participants signed an informed consent form indicating their willingness to participate fully in this study. Additionally, participants were administered a short screening questionnaire designed to assess exclusion criteria previously noted in the literature [28]. Participants were also asked to refrain from tobacco use, eating, and consuming caffeine for the 12 hours prior to data collection [37]. A self-report questionnaire was used to obtain information on age, race, height, gender, relationship status, and other demographic information about these participants. The participants were randomly assigned to either the negative (n = 36) or positive priming group (n = 34). The average Body Mass Index (BMI) for this study sample was 26.48 (*SD* = 6.10), which is considered “Overweight” according to the Centers for Disease Control and Prevention (CDC). It must be noted that the BMI categories were constructed after the study’s manipulations for the sole purpose of testing the hypothesis that there would be differences in survey responses based upon this factor. In this study, participants were not divided into categories prior to the performance of any manipulation. Participants all experienced the exact same stressor (introduction of the vignette) and the only difference between group assignment was whether or not they viewed positive or negative images. Each participant received either course credit or extra credit depending on professor preference for their participation. The experiment was a 2 (priming group: positive vs negative) x 2 (time condition: pre-vignette vs post-vignette) x 2 (sex: male vs female) factorial study design. All studies reported in this manuscript were carried out in accordance with the NIH guidelines and approved by the University of Tennessee Institutional Review Board. This study was conducted during the 2014–2015 academic year.

### Measures

#### Fat phobia scale (short form)

We used this scale to assess attitudes and stereotypical feelings toward individuals with obesity. This 14-item short form of the original 50-item scale was developed by Bacon, Scheltema, and Robinson [38] to study, measure, and treat fat phobic attitudes, fat prejudice and body image, and stigmatization caused by obesity. Responses range from 1 (having the least fat phobic attitudes) to 5 (having the most fat phobic attitudes). Higher total scores suggest higher fat phobic attitudes. Bacon et al. [38] reported a Cronbach’s alpha of 0.87 in their 1984–1991 sample, and a Cronbach’s alpha of 0.91 in their 1999 sample. Both samples consisted of predominately White adults. The current study had a Cronbach’s alpha of 0.75.

#### Perceived stress scale

We incorporated this scale in an attempt to examine participant’s current perceived life stress. This scale was developed by Cohen, Kamarck and Mermelstein [39] to assess global non-specific stress levels during the last month. This survey is comprised of 14-items, of which 7 are positively formulated (i.e. “In the last month, how often have you felt things are going your way?”), and 7-items which are negatively formulated (i.e. “In the last month, how often have you felt that you were unable to control the important things in your life?”). This is a widely used instrument, and higher scores indicate greater stress levels. Coefficient alpha reliability for this measure was 0.84, 0.85, and 0.86 in a test of three student samples [39]. This study reported a Cronbach’s alpha of 0.70 for this sample of college students.

### Other scales included for exploratory purposes

Participants were administered other surveys for exploratory purposes to test potential mediators of the stress effects of exposure to stigmatized others. For example, participants higher in perceived stress or need to belong may show greater responses to the images.

#### Need to belong scale

We used this scale to gauge social factors that could influence participants’ social stress. This scale assesses the degree to which respondents desire to be accepted by others, seek opportunities to belong to social groups, and react negatively when they are shunned, rejected, or ostracized [40]. This is a 10-item scale, and participants will respond on a 5-point scale ranging from “strongly disagree” (1) to “strongly agree” (5). Higher scores indicate a greater need to belong. Mellor and colleagues [41] reported a Cronbach’s alpha of 0.78 in an adult Australian sample. In the current college student sample, a Cronbach’s alpha of 0.60 was observed.

#### Rosenberg self-esteem scale

We employed this scale to measure self-esteem of our participants prior to study manipulation. This is a popular scale developed by Rosenberg [42]. This scale consists of 10 items. The scale ranges from 0 to 30, and scores between 15 and 20 are within normal range. Scores lower than 15 are considered indicative of low self-esteem. This study reports a Cronbach’s alpha of 0.60.

### Physiological measures

We also examined stigma in terms of Autonomic Nervous System (ANS) responses. More specifically, we targeted the Sympathetic Nervous System (SNS) and the stress response resulting from SNS activation. In order to assess the psychosocial stress analyses (vignette) in a more immediate manner, we obtained samples of saliva for alpha amylase. Arousal, a direct result of SNS activation, is measured by skin conductance. Therefore, we included a measure of skin conductance in our study as well.

#### Salivary alpha amylase (sAA)

Alpha Amylase was obtained from saliva samples. More specifically, the “pool-and-drool” technique of salivary data collection was employed. Participants were asked to salivate into a sanitized 50 mL test tube for pre- and post- stressor analyses. sAA concentrations were determined via an assay kit (Salimetrics, State College, PA) expressed in U/mL. Higher levels of sAA are indicative of higher levels of SNS activity. sAA has been used as an alternative way to measure levels of stress in the body, as the Sympathetic-Adrenal-Medullary (SAM) system is activated in short term fight or flight responses to stressful stimuli [43,44]. In relation to psychological stress, sAA is used as a marker to examine adrenergic activity [45]. Rohleder and colleagues [27] examined stress-induced increases of sAA using a psychosocial stress test known as the Trier Social Stress Test (TSST). The authors concluded that acute psychosocial stress, does in fact, increase levels of sAA. Increases in sAA have been observed immediately upon introduction of stress [28]. For this reason, we employed sAA in our study as a more immediate indicator of social stress assessed via SNS activity.

#### Skin conductance

Skin conductance (SC), another indicator of SNS activity, was measured using cuffs with sensors placed on the middle and index fingers of the non-dominant hand. These sensors were connected to the ProComp Infinity (Thought Technology Ltd., CA) analog-to-digital converter. Five-minute pre- and post- stressor recordings were taken and participants were asked to relax during this process. SC is measured in micro-Siemens units. A higher level of skin conductance indicates higher levels of arousal. SC has been used to examine the autonomic nervous system response to stress and other psychophysiological stimuli [46]. Many studies have been conducted which indicate the effectiveness of SC as a good measurement tool for SNS activity. Bursts of sympathetic nervous system (SNS) activity lead to changes in SC [47]. Increases in SC, or Galvanic Skin Response (GSR), are positively correlated with increases in levels of stress as demonstrated by Perala & Sterling [48]. Similarly, Sharma et al. [49] demonstrated that mean GSR increases with the introduction of stress. Therefore, we can infer a link between SC and the stress response.

### Procedure

The experiment was a 2 (priming group: positive vs negative) x 2 (time condition: pre-vignette vs post-vignette) x 2 (sex: male vs female) factorial study design. It must be noted that sAA exhibits a diurnal circadian rhythm and thus variations could be seen in flow rate or concentrations of sAA if not controlled for time of day [45]. To avoid the potential effect of circadian variation, in this study, we examined delta change. The time difference between pre- and post- measures are half an hour, so delta change values from individual to individual are not affected by circadian variation being that we are conducting within-group comparisons of sAA. Therefore, the effects observed in this study were not due to differences in circadian variation.

A document of informed consent was presented on a desktop computer using an online survey generator (i.e. Qualtrics). After providing their consent to the study, the participant was asked to complete all of the surveys on the computer. The participant was then guided into a separate examination room where they were first asked to step onto the scale to assess body weight and then asked to render a saliva sample, which was used to assess baseline sAA levels. First, participants were asked to rinse their mouths with a few ounces of water that was provided to them in a small Dixie cup. This was to ensure no extraneous particles in the mouth would interfere with the analyses. Next, the participant was asked to relax and sit quietly in a chair for ten minutes while allowing the saliva to pool in their mouth and also to avoid sample dilution before the saliva collection [37]. Then, all participants were asked to salivate into a sanitized 50 ml collection tube. All participants were specifically instructed not to spit into the tube as spitting may alter the amount and quality of the enzyme being collected. This “pool-and-drool” process took place three times, and the collection time was approximately five minutes. Samples were centrifuged and further distributed into microtubes and stored at -70°C until subsequent analysis.

In this experimental room, the participants were also asked to relax in a chair for five minutes, while being attached to the encoder (Thought Technology Ltd. Software) where subsequent SC was measured. For skin conductance, the electrode strap was fastened around both fingers on the non-dominant hand tightly enough that the electrode surface was in contact with the finger pad, but not so tightly that it limited blood circulation. The leads were connected to the multichannel Procomp Infiniti hardware and Biograph software from Thought Technology (Montreal, Canada). SC data was collected at 256 Hz. After five minutes of data collection, the sensors were turned off and the participants were exposed to the priming stimuli based upon their previously assigned group.

Regardless of group, all participants were exposed to pictures of obesity in society. Various pictures were obtained from the UCONN Rudd Center’s website. These pictures have been identified as positive images of obese adults and youth engaging in healthy behaviors. These pictures were paired with Google images and given to trained raters to decide which ones were “negative” and “positive” in nature. The validation group consisted of nine raters who viewed over 75 positive and 75 negative images on a computer. The validation group was asked to rate each picture on a -3 to 3 scale from negative to positive. The top 21 images in both categories were chosen for this study. After validation of positive and negative images by nine raters, the pictures with the highest polarized ratings were the ones chosen for this study. The negative group was shown pictures that represented negative societal views of obesity (e.g. obese individuals sitting on a couch watching TV, obese individuals eating profusely, etc.). The positive group was shown pictures that represented a more hopeful positive societal view of obesity (e.g. obese individuals working out, plus size models, fat loss transformations, etc.). Both groups were shown 21 pictures via slideshow on a laptop for about five seconds per picture. This procedure was repeated for a total of two viewings. This exposure time is consistent with previous studies examining emotional induction due to visual stimuli [50].

After exposing all participants to pictures of individuals with obesity, a vignette was introduced. The same vignette was used for both groups. The vignette was given to the participants to read in which an airline kicked an obese individual off of the plane because the individual was too large and could not fully fit in the seat. This vignette is a real-life scenario of discrimination that many people can actually relate to. The vignette served as the stressor in this study. Immediately after the vignette, the participant was asked to answer six brief questions designed to discover whether the positive or negative pictures influenced participants’ attitudes toward the stigmatized obese individual. Questions such as: “Was this individual wronged?” and “Who is at fault, the airline or the obese individual?” were asked. These questions were open-ended in nature, and all participants wrote down their reaction to this vignette.

A second saliva sample for alpha amylase was obtained via the “pool-and-drool” technique. Similar to the first collection process, the participant rendered a three-minute sample of saliva. Finally, another five-minute baseline measure of SC was obtained for each participant. Upon completion of this task the participant was debriefed, thanked for their time, and their participation was complete.

### Data analyses

All data were analyzed using SPSS version 22 (SPSS, Chicago, USA). The hypothesis testing included paired samples T-test, correlations analysis, chi-squared analysis, and repeated measures analysis of variance (ANOVA). In order to test the hypothesis of differences between BMI groups on self-report measures, a t-test was performed. A 2 (priming group) x 2 (Time condition) x 2 (Sex) repeated measures ANOVA, as well as a paired samples T-test, were conducted on the physiological measures of sAA and SC. When examining whether or not sex played a key role in determining responding to sAA, an independent T-Test was computed. In order to examine the relationship between sAA and SC, correlational analyses were performed. In order to assess differences in responding based on priming group, Chi Square analyses were performed. The alpha levels were set at 0.05 for all analyses. Mean change scores on the physiological measures were computed by subtracting post-vignette scores from pre-vignette scores.

## Results

### Analysis of self-report measures

Descriptive statistics for the survey questionnaires, physiological measures, and demographic information for this study are presented in Table 1. Results indicated no significant differences between BMI groups on perceived stress, t(68) = -0.246, *p* = 0.807, fat phobia, t(68) = 0.639, *p* = 0.525, need to belong, t(68) = 0.498, *p* = 0.620, and self-esteem, t(68) = 0.217, *p* = 0.829. For exploratory purposes, we also examined whether there were differences between the priming groups in terms of the self-report measures (see Table 2). This was done to identify possible extraneous differences among the groups prior to the manipulation that could confound the results. Only the self-report measure of Need to Belong showed a statistically significant difference among the priming groups, t(68) = -2.45, *p* = 0.02 (Table 2). To examine the association between the self-reported measures and BMI, a correlational analysis was computed. This correlational analysis revealed a positive correlation between perceived stress and fat phobia (r = 0.33, *p* < 0.01) and negative correlations between perceived stress and self-esteem (r = -0.40, *p* < 0.01), self-esteem and fat phobia (r = -0.46, *p* < 0.01), and self-esteem and need to belong (r = -0.33, *p* < 0.01). However, there was no significant relationship between the self-report measures and BMI.

**Table 1:** Descriptive statistics for the survey questionnaires, physiological measures, and demographics.

Dependent Measures	Mean	Std. Dev.
**PSS**	34.24	4.35
**Fat Phobia**	2.99	0.75
**NTB**	32.71	3.82
**SE**	14.09	2.19
**sAApre**	18.89	31.94
**sAApost**	23.49	36.10
**SCpre**	3.18	2.24
**SCpost**	2.60	2.53
**Age**	20.94	6.11
**BMI**	26.48	6.10

Abbreviations: PSS: Perceived Stress Scale; NTB: Need to Belong; SE: Self-Esteem; sAApre: salivary Alpha-Amylase pre-stressor; sAApost: salivary Alpha-Amylase post-stressor; SCpre: Skin Conductance pre-stressor; SCpost: Skin Conductance post-stressor; BMI: Body Mass Index.

**Table 2:** Independent samples T-test of priming group on dependent measures.

Priming Group						
	Positive		Negative			
Surveys	Mean	SD	Mean	SD	t	*p*
**Self-Esteem**	13.91	2.38	14.25	2.00	0.64	0.52
**Need to Belong**	33.82	3.75	31.67	3.63	-2.45	0.02*
**Fat Phobia**	3.01	0.67	2.97	0.82	-0.22	0.82
**Perceived Stress Scale**	34.24	3.81	34.25	4.85	0.01	0.99

* = p ≤ 0.05.

### Analysis of physiological measures

#### Relationship between sAA and SC

Correlational analyses revealed positive relationships between sAA pre- and post- vignette (r = 0.74, *p* < 0.01) and SC pre- and post- vignette (r = 0.84, *p* < 0.01). Interestingly, these analyses also revealed a positive linear relationship between sAA pre- and SC post- vignette (r = 0.28, *p* < 0.05).

#### Salivary alpha amylase (sAA)

**Fig 1:**
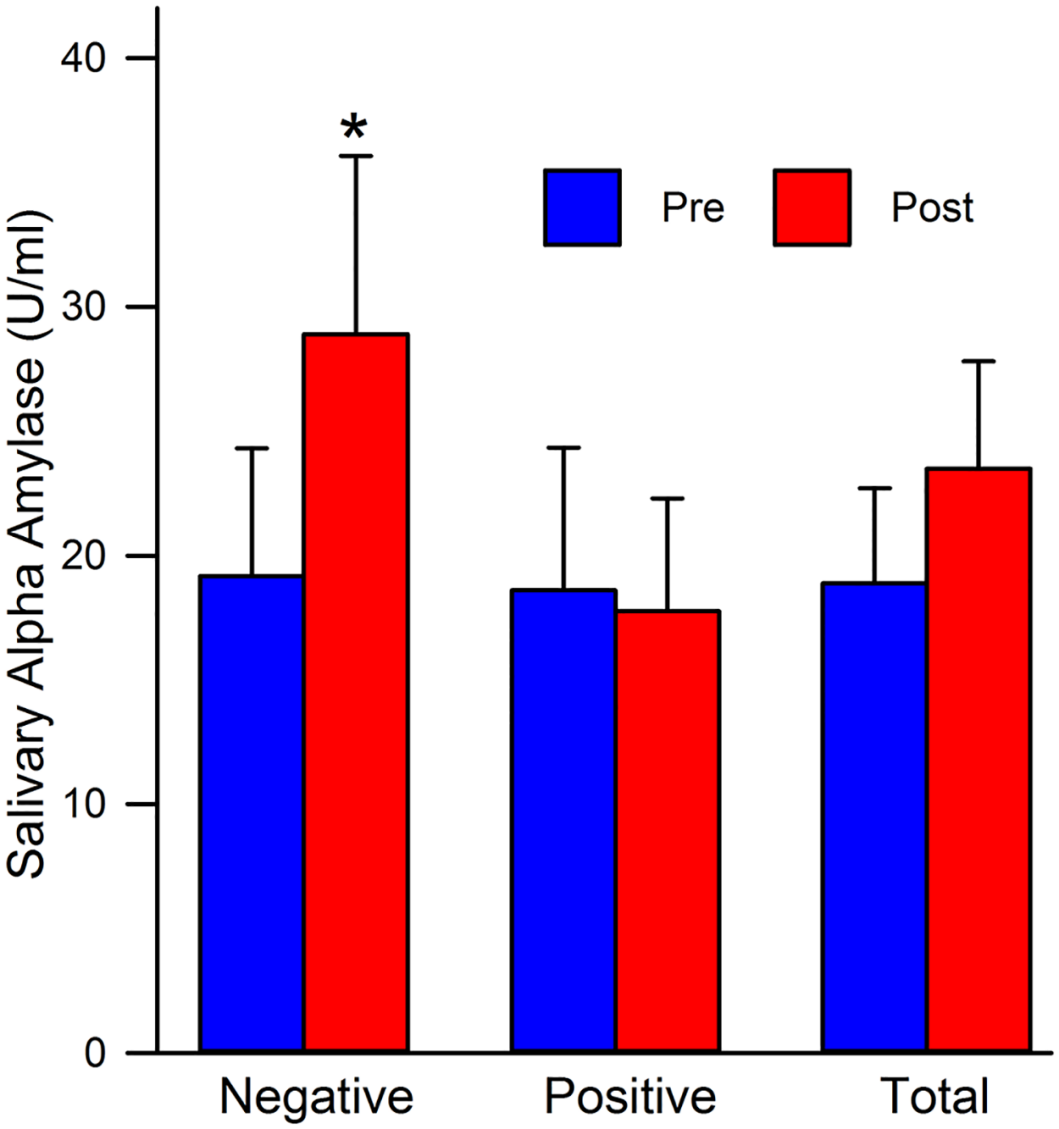
Mean sAA level comparisons by priming group. Negative: all males and females assigned to the negative priming group; Positive: all males and females assigned to the positive priming group; Total: all males and females collapsed across priming groups; Pre: pre-stressor means; Post: post-stressor means. * = *p* ≤ 0.05.

Means and Standard Deviations are provided in Table 3. When testing for between-subject effects, the repeated measures ANOVA revealed no significant main effect of priming, *F*(1, 66) = 0.37, *p* = 0.55, sex, *F*(1, 66) = 0.30, *p* = 0.59, nor the interaction between priming and sex, *F*(1, 66) = 0.03, *p* = 0.87. When testing for within-subject effects, there was no significant main effect of time, *F*(1, 66) = 2.43, *p* = 0.12. Also, the interaction between time and sex, *F*(1, 66) = 0.12, *p* = 0.73, and the 3-way interaction between time, priming, and sex yielded no significant results, *F*(1, 66) = 2.09, *p* = 0.15. However, the analysis did indicate a significant time x priming interaction, *F*(1, 66) = 5.18, *p* = 0.03. When examining the means, the negative priming group responded with higher sAA levels post-vignette than did the positive group (see Fig 1). There was a significant increase in sAA post-vignette in both men and women, t(35) = -2.82, *p* = 0.01. When collapsing across priming group and examining sAA as a function of time, there was a positive linear trend in the change scores between sAA pre- and post-vignette, *F*(1, 69) = 3.32, *p* = 0.07.

**Table 3:** Means and Standard Deviations of sAA & SC by priming group.

	sAA				SC			
	Pre		Post		Pre		Post	
Priming Group	Mean	SD	Mean	SD	Mean	SD	Mean	SD
**Negative**	19.17	30.93	28.90*	42.97	3.32	2.38	2.49**	2.41
**Positive**	18.60	33.45	17.76	26.49	3.03	2.12	2.73	2.68
**Total**	18.89	31.94	23.49	36.10	3.18	2.24	2.60**	2.53

Abbreviations: sAA: salivary Alpha-Amylase; SC: Skin Conductance; pre: pre-stressor means; post: post-stressor means.

* = *p* ≤ 0.05,

** = *p* ≤ 0.01.

#### Skin conductance (SC)/ Galvanic Skin Response (GSR)

**Fig 2:**
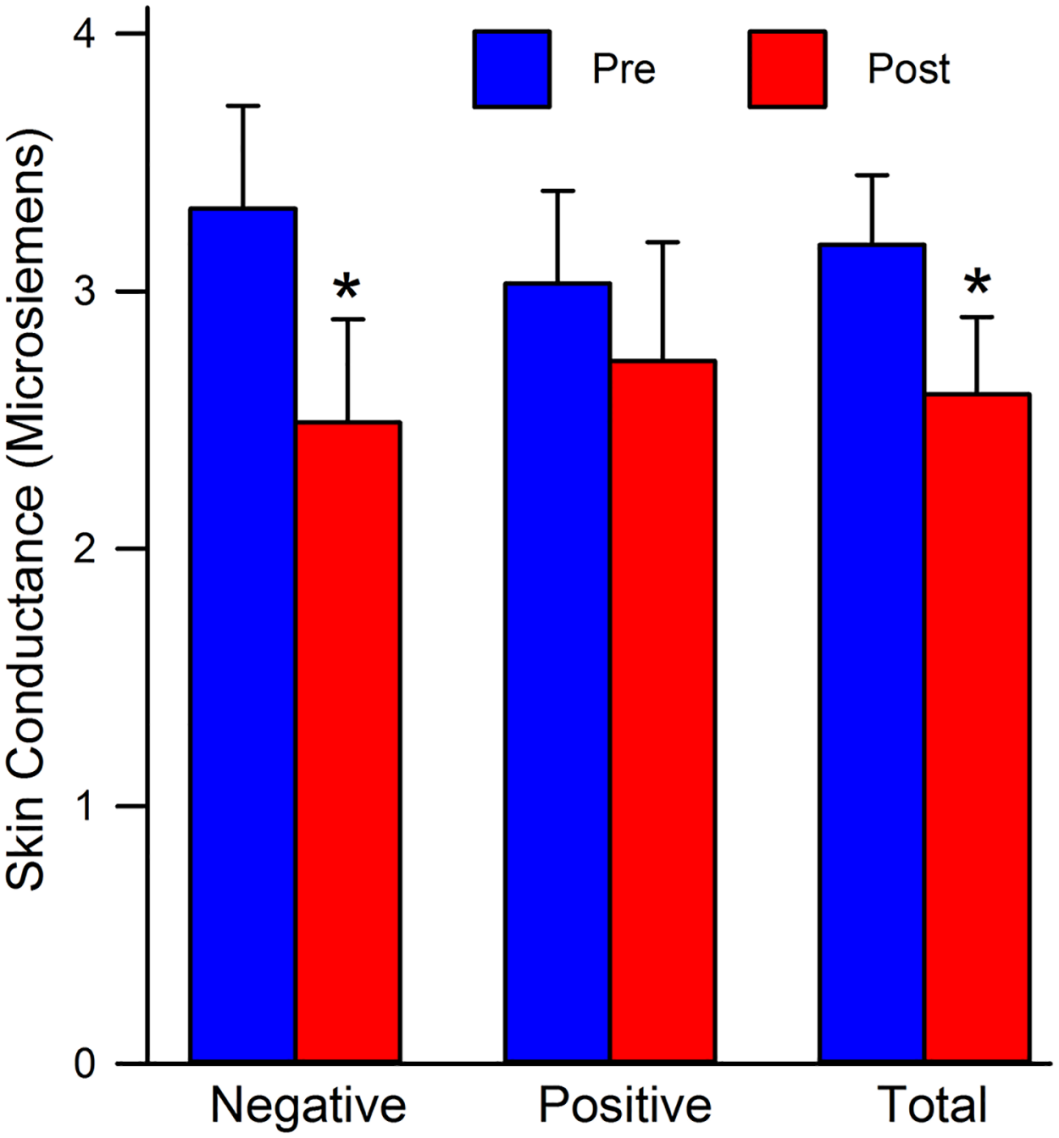
Mean SC level comparisons by priming group. Negative: all males and females assigned to the negative priming group; Positive: all males and females assigned to the positive priming group; Total: all males and females collapsed across priming groups; Pre: pre-stressor means; Post: post-stressor means. * = *p* ≤ 0.01.

When testing for between-subject effects, the repeated measures ANOVA revealed no significant main effect of priming, *F*(1, 66) = 0.13, *p* = 0.72, sex, *F*(1, 66) = 0.35, *p* = 0.56, nor an interaction between priming and sex, *F*(1, 66) = 0.89, *p* = 0.35. When testing for within-subject effects, there was a significant main effect of time, *F*(1, 66) = 7.24, *p* = 0.01. When holding group and sex constant, all participants exhibited a decrease in SC post-vignette compared to pre-vignette (see Fig 2). The interaction between time and priming was significant, *F*(1, 66) = 7.06, *p* = 0.01. When collapsing across groups, both priming groups showed a decrease in SC post-vignette. When examining differences by group, there were no significant differences among the participants in the positive group (t = 1.47, *df* = 33, *p* = 0.15). However, the negative priming group showed a significant decrease in SC post-vignette, t = 3.40, *df* = 35, *p* < 0.01.

The 3-way interaction between time, priming, and sex yielded significant results as well *F*(1, 66) = 6.60, *p* = 0.01. Although both priming groups experienced decreases in SC post-vignette, those in the negative priming group experienced the greatest decreases in SC when compared with their positive group counterparts (see Table 3). Females also exhibited a significant decrease post-vignette, whereas their male counterparts did not (see Tables 4 and 5). Males in the negative priming group pre-vignette exhibited the highest SC reactivity, while females in the negative priming group post-vignette exhibited the lowest SC responding. However, the results did not indicate a significant time x sex interaction *F*(1, 66) = 2.71, *p* = 0.10 when collapsing across priming groups. Change scores in SC were not significant *F*(1, 68) = 2.78, *p* = 0.10. However, the paired samples t-test indicated that there was a statistically significant difference in the scores for SC pre- (*M* = 3.18, SD = 2.25) and post-vignette (*M* = 2.60, SD = 2.53) collapsed across condition, t(69) = 3.53, *p* < 0.01.

**Table 4:** Means and Standard Deviations of sAA & SC by sex.

	sAA				SC			
	Pre		Post		Pre		Post	
Sex	Mean	SD	Mean	SD	Mean	SD	Mean	SD
**Female**	20.57	29.14	24.55	36.33	3.16	1.97	2.42*	2.06
**Male**	14.70	38.59	20.81	36.32	3.24	2.88	3.06	3.46
**Total**	18.89	31.94	23.49	36.10	3.18	2.24	2.60*	2.53

Abbreviations: sAA: salivary Alpha-Amylase; SC: Skin Conductance; pre: pre-stressor means; post: post-stressor means.

* = *p* ≤ 0.01.

**Table 5:** Paired samples T-tests of sAA and SC.

	sAA (pre-post)				SC (pre-post)			
Sex	Mean	SD	t	*p*	Mean	SD	t	*p*
**Female**	-3.98	21.68	-1.30	0.20	0.74	1.14	4.55	0.00**
**Male**	-6.12	31.49	-0.87	0.40	0.18	1.76	0.45	0.66

Abbreviations: sAA (pre-post): salivary Alpha-Amylase pre-stressor means minus post-stressor means; SC (pre-post): Skin Conductance pre-stressor means minus post-stressor means.

** = p ≤ 0.01

### Analysis of sex differences on the dependent measures

#### Salivary alpha amylase (sAA)

**Fig 3:**
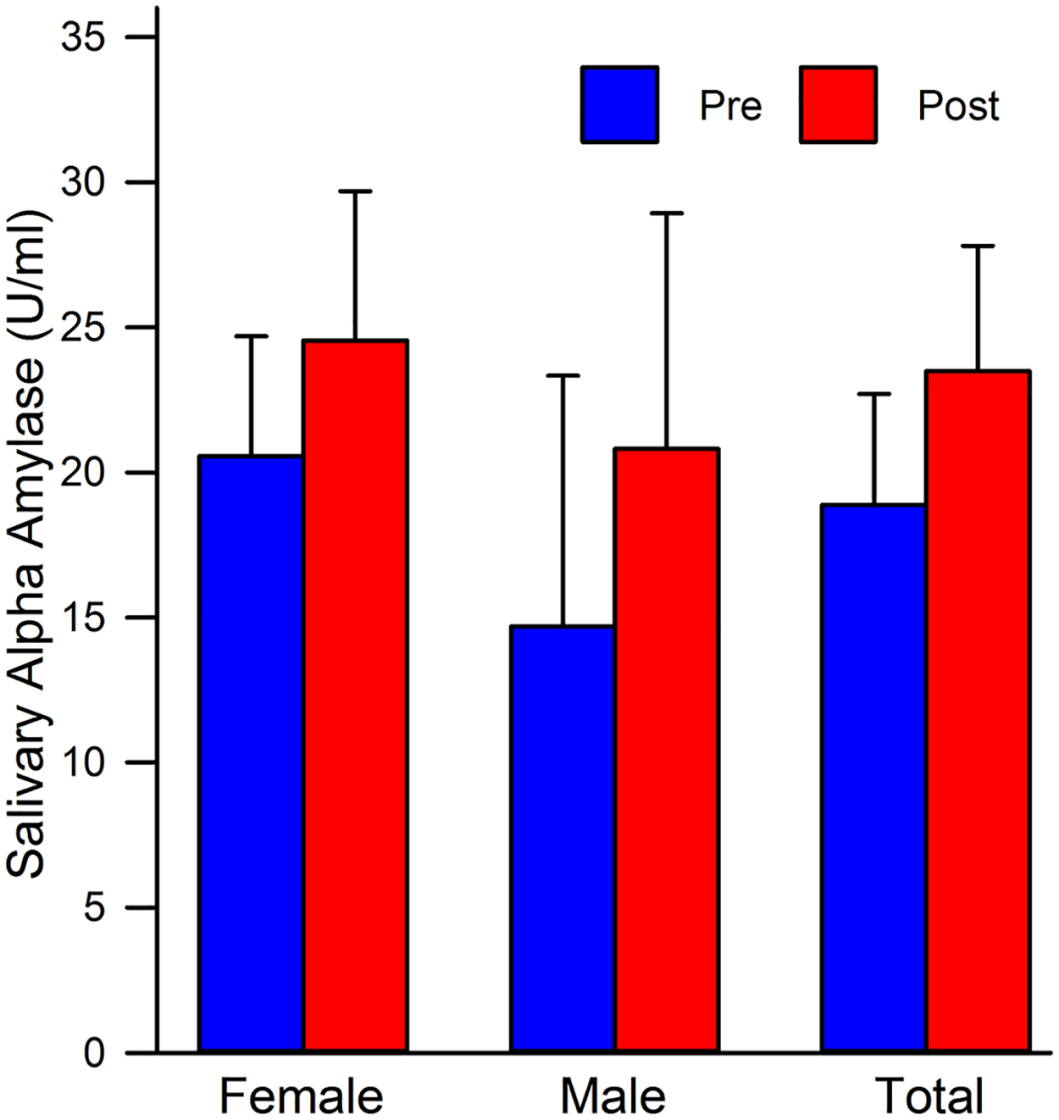
Mean sAA level comparison by sex. Female: all females collapsed across priming groups; Male: all males collapsed across priming groups; Total: all males and females collapsed across priming groups; Pre: pre-stressor means; Post: post-stressor means.

When examining whether or not sex played a key role in determining responding to sAA, it was concluded that when collapsed across priming conditions and looking at all of the participants as a whole, sex was not significant t(69) = 1.56, *p* = 0.12. There were also no significant differences in sAA response between pre- to post-vignette in both women t(49) = 1.30, *p* = 0.20 and men t(19) = 0.87, *p* = 0.40 when holding the other sex constant (see Fig 3). Overall, men had lower sAA both pre- and post-vignette compared to women (see Table 4).

#### Skin conductance (SC)/ Galvanic Skin Response (GSR)

**Fig 4:**
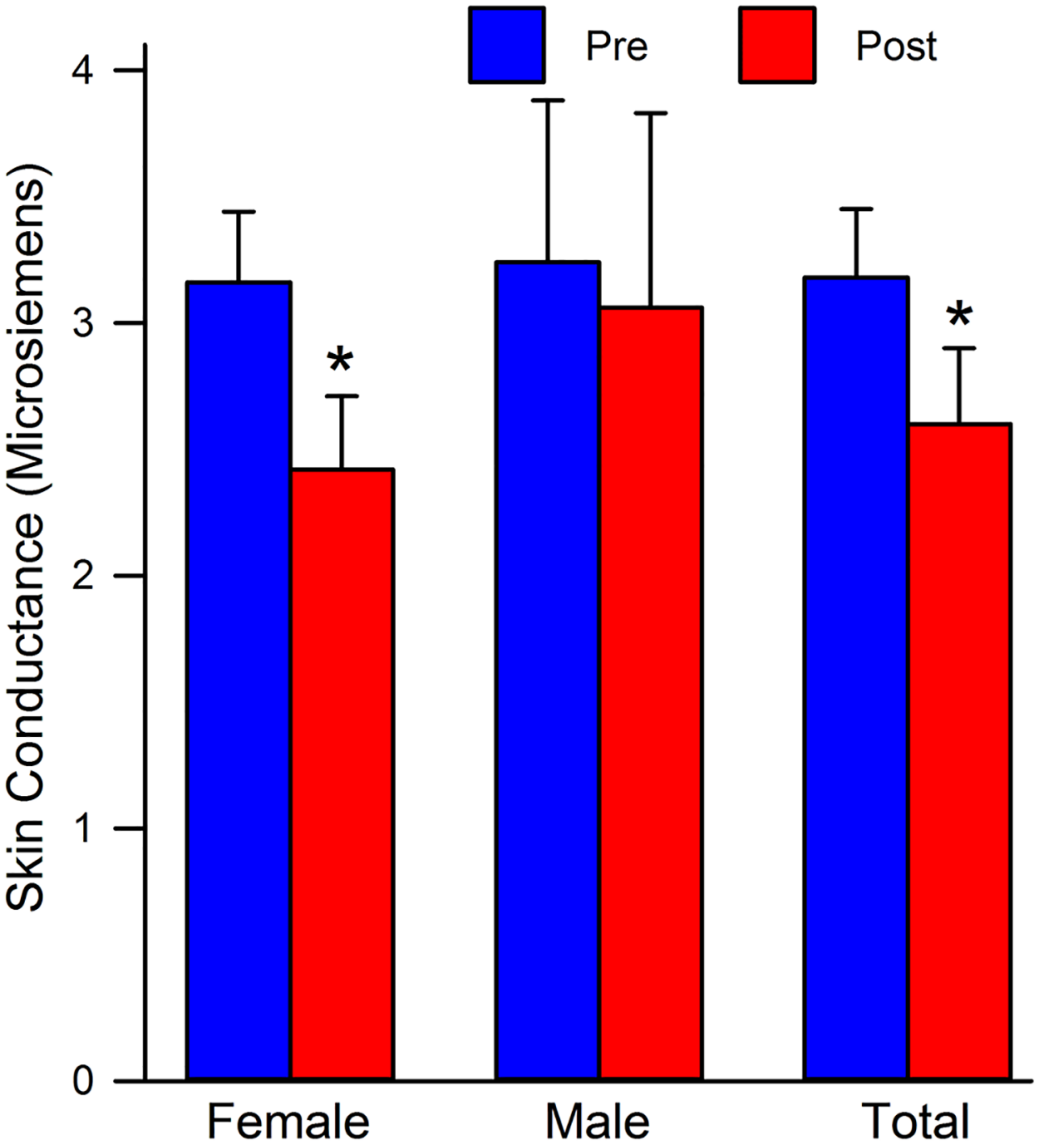
Mean SC level comparison by sex. Female: all females collapsed across priming groups; Male: all males collapsed across priming groups; Total: all males and females collapsed across priming groups; Pre: pre-stressor means; Post: post-stressor means. * = *p* ≤ 0.01.

In terms of SC, there was a significant main effect of sex in regard to time, t(69) = 3.53, *p* < 0.01. When collapsing across priming groups, both males and females experienced a decline in SC. When parceling out to see where the significance lies, we found that there were no differences in SC for males, t(19) = 0.45, *p* = 0.66. However, for females there was a significant decrease in SC post-stressor, t(49) = 4.55, *p* < 0.01 (see Fig 4).

### Analysis of priming manipulation

In order to address the question of whether or not priming affected participants’ view of discrimination, qualitative responses to the vignette had to be coded. Items were coded on attribution of blame to either the airline, obese individual, or both. The question of “who was at fault?” was asked of the participants. When conducting the chi-square analyses to examine the degree of attribution of blame for the post-vignette questions, no significant attribution of blame being placed on the airline [χ^2^ = 3.32, *p* = 0.07], nor the obese individual [χ^2^ = 0.30, *p* = 0.58] was found as the result of priming. Both groups were fairly equal on all response items (see Table 6).

**Table 6:** Chi Square results for all vignette questions.

Vignette Questions	Chi-square	Df	*p*
**Individual Wronged**	1.15	2	0.56
**Extent Wronged**	2.20	2	0.33
**Attributional Blame**	3.32	2	0.19
**Extent Blame on Individual**	0.30	1	0.58
**Extent Blame on Airline**	3.32	1	0.07

## Discussion

The primary purpose of this study was to examine whether or not the priming of individuals to either positive or negative images of obesity would influence subsequent responding to an actual obesity discrimination incident in the media. Principle findings of this study were: 1) an increase in sAA existed when exposed to the psychosocial stressor across both priming groups, 2) a time by priming interaction yielded differences in sAA responding, 3) differences in SC between pre and post measures, 4) the interaction between time and priming with regard to SC, 5) a time by priming by sex interaction in terms of SC, 6) the priming manipulation was ineffective in polarizing the way individuals attributed blame, and 7) a main effect of sex was present for SC, but not for sAA.

It was hypothesized that there would be differences between individuals classified as overweight and normal BMI on the self-report measures. In terms of BMI classification, there were no significant differences on the measures of perceived stress, fat phobia, need to belong, or self-esteem. Differences in perceived stress were expected based upon a study by Farag et al. [51] in which higher levels of perceived stress were correlated with higher salivary cortisol levels and BMI. Previous research indicated that individuals with obesity also tend to exhibit decreased levels of self-esteem [52]. However, our findings are consistent with that of Dutton et al. [53] in which we found no relationship between need to belong and BMI. One reason why we did not observe the expected results in this study could be that higher levels of BMI are more prevalent in today’s society, specifically in the Southeast. Individuals may not feel as though they are inherently different from others around them. Thus, they do not feel as ostracized, nor exhibit lower self-esteem. It must be noted that the participants in this current study displayed a mean BMI that is considered overweight. More importantly, our findings are consistent with Hayran et al. [54] who also reported no significant differences in fat phobic attitudes as a function of BMI. A more diverse sample in regard to weight may be necessary to explore these psychological differences.

Our second hypothesis was that there would be differences between the positive and negative obesity primed groups on the physiological measures. In terms of sAA, psychosocial stress has been shown to induce increases in this salivary enzyme [55]. The analyses revealed no differences in a main effect of priming, sex, or time. Neither the priming by sex, time by sex, nor priming by sex by time interactions showed significant results. There are several reasons for the null findings regarding sAA. It could be that our sample size was too small to detect such changes, the priming manipulation was not strong enough, and/or that the stressor was not sensitive enough to evoke noticeable changes. However, there was a significant priming by time interaction.

The negative priming group at time 2 (post-vignette), exhibited the highest overall response, indicating greater reactivity to the vignette. This could be because individuals in the negative group may have experienced negative thoughts about the obese images prior to reading the vignette. Thus, upon reading the vignette and imagining the embarrassment or discomfort the obese individual may have felt after being kicked off of the airline, participants in this group may have experienced more stress. An increase in sAA at time 2 alludes to the notion that the vignette was successful in evoking stress in all participants. This psychosocial stressor consequently induced activation of the sympathetic nervous system causing the increase in sAA. Finally, there was a trend in the hypothesized direction in terms of change scores from pre to post-vignette for sAA. After the introduction of the vignette, sAA increased at time 2, relative to time 1, when collapsed across priming groups. This increase in sAA is consistent with previous literature in that the introduction of a psychosocial stressor has been used to evoke increases in sAA when stressed [28]. This increase is indicative of something that is uniform in both conditions, such as the introduction of the vignette. This could play a role in sAA responding that differs depending on the time sAA was obtained. sAA peaks during psychological and/or psychosocial stressful situations [56]. Being that this vignette incorporated a real-life stressful situation, evocation of sAA may be imminent. Furthermore, sAA appears to be a good indicator of the effectiveness of positive priming. When comparing means for the positive and negative priming groups pre-stressor, they are fairly identical; however, after introduction of the priming manipulations, they differ drastically. This seems to allude to the notion that positive priming may serve as a buffer for sympathetic nervous system activation as the stressful encounter was not as pronounced in this group.

When examining SC, there was a significant decrease in arousal between time 1 and time 2. The introduction of the vignette could be an explanation for such changes. SC was obtained at time 1 prior to the vignette, as well as, time 2, after the vignette. The vignette appears to have played an important role in the physiological arousal of the participants that manifested itself via SC response. Even though both groups showed a decline in SC in response to the vignette, the negative group was the one most affected by the priming manipulation. Significant decreases were shown within the negative group manifesting itself post-vignette. This seems to suggest that those individuals within the negative priming group were less aroused by the vignette than their counterparts. It is plausible that the negative priming facilitated a blunted affect to the vignette in these participants. However, the positive priming may have facilitated an unexpected affect in participants, which lead to less of a decline in SC. Our findings are similar to Graves, Cassisi, & Penn [57] in which a dampening of negative affect and Skin Conductance were associated with reductions to stigma of schizophrenia.

Interestingly, there was a significant 3-way interaction on SC reactivity. Males in the negative priming group pre-vignette exhibited the highest SC reactivity, while females in the negative priming group post-vignette exhibited the lowest SC responding. SC is used to assess arousal. In this sample of college students, males and females reported similar levels of arousal prior to the introduction of the vignette. This finding is expected as no experimental manipulation has occurred thus far. However, after introducing the vignette, females decline drastically in their SC responding compared with males. This is indicative of sustained arousal in the males and decreased arousal in the females. This could either suggest that our sample of men were more sympathetic to the stigmatization scenario or that body image issues tend to be arousing to them. On the other side of this interesting finding, is the fact that women in our sample dropped significantly in SC arousal. This could be due to a number of factors. First, women in general may become conditioned to dealing with weight issues, and our scenario does not affect them as much. Therefore, reducing their arousal levels to the vignette due to repeated exposure to similar situations. Secondly, our female participants may not have viewed the obese images as threatening and therefore were less aroused. Furthermore, compared to the women in the negative priming manipulation, the female participants in the positive priming group were not aroused. This may be due to feelings of satisfaction, which were sparked by the positive images.

When examining mean change scores, we did see that the negative stigma group exhibited significantly less SC responding compared to the positive priming group. This blunted response could be due to the fact that this group was already primed to negativity pre-vignette and therefore did not experience the same arousal, as did those who were primed in a more positive manner. This finding is consistent with the previous literature, in that acute psychological stress has been shown to cause blunted physiological responding [58]. However, a more plausible explanation is that of cognitive dissonance. Individuals in the positive priming group were confronted with images that may have conflicted with their preconceived ideas on obesity, thus causing an increase in SC.

Our third hypothesis was that the priming manipulation would have an influential effect on participants’ view of discrimination. Priming has been shown to induce differences based upon group assignment [59]. After coding the responses, it was concluded that there were no differences in response to the vignette. We expected that the priming manipulation would contribute to either more support and less attribution in the positive group or more attribution and less support for the obese in the negative group. The results indicated that regardless of group, most attributed blame to the airline company and not to the individual. Although it is good that there was less individual attribution overall, it does not support our hypothesis that the priming manipulation would influence this attribution of blame. Our findings contradict Crocker and Major (2003) who suggest that perceived controllability and visibility of the stigmatized condition are important determinants of targeted stigmatization [60]. This particular vignette may have elicited feelings of empathy given that the obese individual was potentially publicly humiliated by being asked to exit the flight because she was considered “too fat to fly”. As stated earlier, our sample was overweight and primarily from the southeast. According to the Centers for Disease Control and Prevention, prevalence rate of self-reported obesity among adults in the south is very high. In support of this explanation, we found the attitudes about obesity in our sample tend to be more positive in general. That in and of itself is a great finding. Future research on this topic should examine populations in other regions of the United States such as the west coast or even in the northern states to see if this trend still holds.

Our fourth and final hypothesis was that there would be sex differences on the dependent measures of sAA and SC. In terms of sAA, males and females did not differ as a function of time. Both males and females responded similarly in both situations pre- and post-vignette. Both groups experienced an increase in sAA in response to the vignette, which was in the hypothesized direction. In terms of SC, sex played an important role in responding. Females decreased significantly more than their male counterparts post-vignette. As mentioned previously, this finding could be due to a developed blunted affect that manifested itself in our sample of college-aged women. In the current study, the men were essentially equally aroused pre- and post-vignette. This finding could lend contradiction to the previously assumed norms in the literature that men do not care about body image [61]. However, men may still not care about their body image, but may be interested in the image of others, which in this vignette happened to be a female. In conclusion, sex was only a factor in SC responding.

It is important to note that there was no clear reciprocal correlation between sAA and SC for both pre- and post-vignette. However, sAA pre and SC post were significantly correlated and in the same positive direction. This is an important finding as it is indicative of differing mechanisms between the two physiological measures that may be driving these results. We posit that sAA and SC may have differing reaction times to social stressors. sAA is an immediate measure of stress whereas SC tends to vary in its’ time course. Bach, Flandin, Friston, and Dolan [62] reported that the variance in skin conductance could be explained by differing event-related skin conductance responses. Event-related SC responses tend to peak in seconds, whereas non-stimulus-locked SC responses can vary up to minutes. Although the vignette proved to stimulate the sAA response, it may not have been enough to evoke immediate SC responding. This suggests a time parameter continuity in the SNS in which sAA is activated due to an immediate stressor. However, the ruminating effects of the stressor may later activate SC. Future research should assess the differing SC time courses (e.g. last minute of baseline and first minute post vignette) to assess the mechanism of action related to SC and SNS activity in regard to psychosocial stressors.

Several strengths of the current study should be noted. First, we assessed both men and women, whereas much of the existing literature focused solely on women [24,63]. Second, we examined two different physiological modalities responding to psychosocial stress. In regard to sAA, we found that the vignette seemed to evoke stress. SNS activation occurred in response to the social stressor. We found that SC was also a sensitive measure of obesity stigma. There were differences among stigma groups on the SC measure in response to the vignette. We therefore attributed these significant differences to the sympathetic nervous system’s stress response evoked by the vignette. Our results suggest that although seemingly responding in different fashions, both sAA and SC can be utilized in assessments of the SNS’s response to psychosocial stressors, and more specifically, to obesity-related stimuli. We expanded upon the literature via the inclusion of a qualitative analysis component. We reviewed the written responses to attribution of blame and it provided us with insight as to how the participants in our study actually felt about real-life stigma as reported by the media.

A number of study limitations are also present. We aimed to assess both men and women in our study, but there were over twice as many women than men who participated. In order to obtain a more accurate representation, more men need to be recruited to examine potential sex effects. Future studies should include a larger and more diverse sample size in order to increase the generalizability of the findings. It is also possible that due to the order of operations with weight being taken before participants viewing of the images, individuals were inherently more stressed prior to the manipulation. One simple solution to this is that weight could be taken at the close of future studies to eliminate potential confounding results. Another limitation is the chosen vignette utilized in this study. It is possible that the vignette elicited empathy, and therefore lessened the impact of the priming manipulation. The vignette included a woman in the stigmatizing situation. Also, participants read the article and did not witness this event first hand or by visual representation. Future studies should manipulate the gender of the person in the vignette and test whether the delivery of the discrimination influences results. Finally, the priming manipulation may not have been strong enough. Participants were shown either positive or negative images of individuals with obesity and were expected to respond to the vignette accordingly. The positive pictures may not have been “positive” enough and the negative pictures not “negative” enough. Although we had nine people rate the pictures in terms of positive or negative images, with 75% agreement, the pictures may not have been sensitive enough to evoke the response we were looking for. It must be noted that the priming manipulation had an influence on ANS responses, but it was not sensitive enough to change behavioral responses to obesity stigma. Future studies should incorporate a control group in which an attitude assessment is administered before and after the experimental priming manipulation to better assess attitude change.

The current study found college students to be largely unaffected by the positive or negative images of individuals with obesity. Regardless of priming group, participants were less likely to indicate a dispositional attribution to the individual removed from the flight because of weight issues. There was some support for the stressfulness of the vignette used in this study. Participants in the negative prime image condition displayed greater change SC values from Time 1 to Time 2 than those in the positive prime image group. All participants showed an increase in sAA post-stressor as well. This further illustrates the utility of both sAA and SC as dependent measures in stigma related studies.

In conclusion, the media is very powerful. Overweight people remain among the last acceptable target of derogatory behavior in both TV and film [10]. They are commonly seen engaging in stereotypical eating behaviors and are rarely depicted in romantic relationships [64]. Ultimately, and most importantly, in identifying and documenting variables that contribute to the stigma of obesity, adverse health outcomes associated with this form of social stigma can also be reduced. This can in turn contribute to a society with greater mental and psychological well-being in the long run. The current study found that individuals in the negative priming group had significantly more SC arousal and exhibited the highest overall sAA response post-vignette when compared to the positively primed group. Our findings suggest that by reducing negative portrayals of obesity in the minds of individuals in society, we can reduce stigma and the various physiological consequences associated with it. Therefore, clinicians addressing stigma issues should consider the use of positively primed images as a method for reducing the possible long-term physiological consequences of such exposure.

## References

[1] Ogden CL, Carroll MD, Kit BK, Flegal KM. Prevalence of childhood and adult obesity in the United States, 2011–2012. Jama. 2014 2 26;311(8):806–14. doi: 10.1001/jama.2014.732 24570244 10.1001/jama.2014.732 PMC4770258

[2] Ahern J, Stuber J, Galea S. Stigma, discrimination and the health of illicit drug users. Drug and alcohol dependence. 2007 5 11;88(2):188–96. 17118578 10.1016/j.drugalcdep.2006.10.014

[3] Major B, O'brien LT. The social psychology of stigma. Annu. Rev. Psychol. 2005 2 4;56:393–421. doi: 10.1146/annurev.psych.56.091103.070137 15709941 10.1146/annurev.psych.56.091103.070137

[4] Prelow HM, Mosher CE, Bowman MA. Perceived racial discrimination, social support, and psychological adjustment among African American college students. Journal of Black Psychology. 2006 11 1;32(4):442–54.

[5] Puhl RM, Heuer CA. Obesity stigma: important considerations for public health. American journal of public health. 2010 6;100(6):1019–28. doi: 10.2105/AJPH.2009.159491 20075322 10.2105/AJPH.2009.159491 PMC2866597

[6] Gee GC, Ro A, Gavin A, Takeuchi DT. Disentangling the effects of racial and weight discrimination on body mass index and obesity among Asian Americans. American journal of public health. 2008 3;98(3):493–500. doi: 10.2105/AJPH.2007.114025 18235065 10.2105/AJPH.2007.114025 PMC2253588

[7] Sawyer PJ, Major B, Casad BJ, Townsend SS, Mendes WB. Discrimination and the stress response: psychological and physiological consequences of anticipating prejudice in interethnic interactions. American Journal of Public Health. 2012 5;102(5):1020–6. doi: 10.2105/AJPH.2011.300620 22420818 10.2105/AJPH.2011.300620 PMC3483920

[8] Torres L, Ong AD. A daily diary investigation of Latino ethnic identity, discrimination, and depression. Cultural Diversity and Ethnic Minority Psychology. 2010 10;16(4):561 doi: 10.1037/a0020652 21058819 10.1037/a0020652

[9] Verkuyten M. Perceived discrimination and self-esteem among ethnic minority adolescents. The Journal of social psychology. 1998 8 1;138(4):479–93. doi: 10.1080/00224549809600402 9664864 10.1080/00224549809600402

[10] Puhl RM, Heuer CA. The stigma of obesity: a review and update. Obesity. 2009 5 1;17(5):941–64. doi: 10.1038/oby.2008.636 19165161 10.1038/oby.2008.636

[11] Lewis TT, Barnes LL, Bienias JL, Lackland DT, Evans DA, De Leon CF. Perceived discrimination and blood pressure in older African American and white adults. The Journals of Gerontology Series A: Biological Sciences and Medical Sciences. 2009:glp062. 10.1093/gerona/glp062 PMC2720886 19429703

[12] Zeiders KH, Hoyt LT, Adam EK. Associations between self-reported discrimination and diurnal cortisol rhythms among young adults: The moderating role of racial ethnic minority status. Psychoneuroendocrinology. 2014 12 31;50:280–8. doi: 10.1016/j.psyneuen.2014.08.023 25262035 10.1016/j.psyneuen.2014.08.023 PMC4254319

[13] Stuart H. Media portrayal of mental illness and its treatments. CNS drugs. 2006 2 1;20(2):99–106. 16478286 10.2165/00023210-200620020-00002

[14] Hilbert A, Rief W, Braehler E. Stigmatizing Attitudes Toward Obesity in a Representative Population-based Sample. Obesity. 2008 7 1;16(7):1529–34. doi: 10.1038/oby.2008.263 18464749 10.1038/oby.2008.263

[15] Latner JD, Rosewall JK, Simmonds MB. Childhood obesity stigma: association with television, videogame, and magazine exposure. Body image. 2007 6 30;4(2):147–55. doi: 10.1016/j.bodyim.2007.03.002 18089260 10.1016/j.bodyim.2007.03.002

[16] Kim SH, Anne Willis L. Talking about obesity: News framing of who is responsible for causing and fixing the problem. Journal of health communication. 2007 6 14;12(4):359–76. doi: 10.1080/10810730701326051 17558788 10.1080/10810730701326051

[17] Thayer JF, Sternberg E. Beyond heart rate variability. Annals of the New York Academy of Sciences. 2006 11 1;1088(1):361–72. 17192580 10.1196/annals.1366.014

[18] Himmelstein MS, Incollingo Belsky AC, Tomiyama AJ. The weight of stigma: cortisol reactivity to manipulated weight stigma. Obesity. 2015 2 1;23(2):368–74. doi: 10.1002/oby.20959 25522347 10.1002/oby.20959

[19] Papadopoulos S, Brennan L. Correlates of weight stigma in adults with overweight and obesity: a systematic literature review. Obesity. 2015 9 1;23(9):1743–60. doi: 10.1002/oby.21187 26260279 10.1002/oby.21187

[20] Tomiyama AJ. Weight stigma is stressful. A review of evidence for the Cyclic Obesity/Weight Based Stigma model. Appetite. 2014 11 1;82:8–15. doi: 10.1016/j.appet.2014.06.108 24997407 10.1016/j.appet.2014.06.108

[21] Vartanian LR, Smyth JM. Primum non nocere: obesity stigma and public health. Journal of bioethical inquiry. 2013 3 1;10(1):49–57. doi: 10.1007/s11673-012-9412-9 23288439 10.1007/s11673-012-9412-9

[22] Schvey NA, Puhl RM, Brownell KD. The stress of stigma: exploring the effect of weight stigma on cortisol reactivity. Psychosomatic medicine. 2014 2 1;76(2):156–62. doi: 10.1097/PSY.0000000000000031 24434951 10.1097/PSY.0000000000000031

[23] Puhl RM, Brownell KD. Psychosocial origins of obesity stigma: toward changing a powerful and pervasive bias. Obesity reviews. 2003 11 1;4(4):213–27. 14649372 10.1046/j.1467-789x.2003.00122.x

[24] Hebl MR, Turchin JM. The stigma of obesity: What about men?. Basic and applied social psychology. 2005 9 1;27(3):267–75.

[25] Hellhammer DH, Wüst S, Kudielka BM. Salivary cortisol as a biomarker in stress research. Psychoneuroendocrinology. 2009 2 28;34(2):163–71. doi: 10.1016/j.psyneuen.2008.10.026 19095358 10.1016/j.psyneuen.2008.10.026

[26] Jackson SE, Kirschbaum C, Steptoe A. Perceived weight discrimination and chronic biochemical stress: A population-based study using cortisol in scalp hair. Obesity. 2016 12 1;24(12):2515–21. doi: 10.1002/oby.21657 27740706 10.1002/oby.21657 PMC5132135

[27] Rohleder N, Nater UM, Wolf JM, Ehlert U, Kirschbaum C. Psychosocial stress-induced activation of salivary alpha-amylase: an indicator of sympathetic activity?. Annals of the New York Academy of Sciences. 2004 12 1;1032(1):258–63. 15677423 10.1196/annals.1314.033

[28] Nater UM, Rohleder N, Gaab J, Berger S, Jud A, Kirschbaum C, Ehlert U. Human salivary alpha-amylase reactivity in a psychosocial stress paradigm. International Journal of Psychophysiology. 2005 3 31;55(3):333–42. doi: 10.1016/j.ijpsycho.2004.09.009 15708646 10.1016/j.ijpsycho.2004.09.009

[29] van Stegeren AH, Wolf OT, Kindt M. Salivary alpha amylase and cortisol responses to different stress tasks: impact of sex. International Journal of Psychophysiology. 2008 7 31;69(1):33–40. doi: 10.1016/j.ijpsycho.2008.02.008 18417235 10.1016/j.ijpsycho.2008.02.008

[30] Nater UM, Rohleder N. Salivary alpha-amylase as a non-invasive biomarker for the sympathetic nervous system: current state of research. Psychoneuroendocrinology. 2009 5 31;34(4):486–96. doi: 10.1016/j.psyneuen.2009.01.014 19249160 10.1016/j.psyneuen.2009.01.014

[31] Engert V, Vogel S, Efanov SI, Duchesne A, Corbo V, Ali N, Pruessner JC. Investigation into the cross-correlation of salivary cortisol and alpha-amylase responses to psychological stress. Psychoneuroendocrinology. 2011 10 31;36(9):1294–302. doi: 10.1016/j.psyneuen.2011.02.018 21470780 10.1016/j.psyneuen.2011.02.018

[32] Olson MA, Fazio RH. Implicit attitude formation through classical conditioning. Psychological Science. 2001 9 1;12(5):413–7. doi: 10.1111/1467-9280.00376 11554676 10.1111/1467-9280.00376

[33] Kurzban R, Leary MR. Evolutionary origins of stigmatization: the functions of social exclusion. Psychological bulletin. 2001 3;127(2):187 11316010 10.1037/0033-2909.127.2.187

[34] Stephan CW, Stephan WG. Reducing intercultural anxiety through intercultural contact. International Journal of Intercultural Relations. 1992 12 1;16(1):89–106.

[35] Tajfel H, Turner JC. The Social Identity Theory of Intergroup Behavior Psychology of Intergroup Relations. Eds. Worchel S. and Austin WG. Chicago: Nelson.

[36] Myers A, Rosen JC. Obesity stigmatization and coping: relation to mental health symptoms, body image, and self-esteem. International journal of obesity. 1999 3 1;23(3):221–30. 10193866 10.1038/sj.ijo.0800765

[37] Salimetrics, LLC. [Internet]. Saliva Collection and Handling Advice; 2015: https://www.salimetrics.com/assets/documents/Saliva_Collection_Handbook.pdf

[38] Bacon JG, Scheltema KE, Robinson BE. Fat phobia scale revisited: the short form. International journal of obesity. 2001 2 1;25(2):252 doi: 10.1038/sj.ijo.0801537 11410828 10.1038/sj.ijo.0801537

[39] Cohen S, Kamarck T, Mermelstein R. A global measure of perceived stress. Journal of health and social behavior. 1983 12 1:385–96. 6668417

[40] Leary MR, Kelly KM, Cottrell CA, Schreindorfer LS. Construct validity of the need to belong scale: Mapping the nomological network. Journal of personality assessment. 2013 11 1;95(6):610–24. doi: 10.1080/00223891.2013.819511 23905716 10.1080/00223891.2013.819511

[41] Mellor D, Stokes M, Firth L, Hayashi Y, Cummins R. Need for belonging, relationship satisfaction, loneliness, and life satisfaction. Personality and individual differences. 2008 8 31;45(3):213–8.

[42] Rosenberg M. Society and the adolescent self-image. Princeton, NJ: Princeton university press;1965 3.

[43] Ehlert U, Erni K, Hebisch G, Nater U. Salivary α-amylase levels after yohimbine challenge in healthy men. The Journal of Clinical Endocrinology & Metabolism. 2006 12;91(12):5130–3. 16968802 10.1210/jc.2006-0461

[44] Sapolsky R. Taming stress. Scientific American. 2003 9 1;289(3):86–95. 12951832 10.1038/scientificamerican0903-86

[45] Nater UM, Rohleder N, Schlotz W, Ehlert U, Kirschbaum C. Determinants of the diurnal course of salivary alpha-amylase. Psychoneuroendocrinology. 2007 5 31;32(4):392–401. doi: 10.1016/j.psyneuen.2007.02.007 17418498 10.1016/j.psyneuen.2007.02.007

[46] Guyton Arthur C, Hall Jhon E. Textbook of Medical Physiology.

[47] Storm H, Myre K, Rostrup M, Stokland O, Lien MD, Raeder JC. Skin conductance correlates with perioperative stress. Acta Anaesthesiologica Scandinavica. 2002 8 1;46(7):887–95. 12139547 10.1034/j.1399-6576.2002.460721.x

[48] Perala CH, Sterling BS. Galvanic skin response as a measure of soldier stress. ARMY RESEARCH LAB ABERDEEN PROVING GROUND MD HUMAN RESEARCH AND ENGINEERING DIRECTORATE; 2007 May.

[49] Sharma R, Khera SH, Mohan AM, Gupta N, Ray RB. Assessment of computer game as a psychological stressor. Indian journal of physiology and pharmacology. 2006 10 4;50(4):367 17402266

[50] van Stegeren A, Rohleder N, Everaerd W, Wolf OT. Salivary alpha amylase as marker for adrenergic activity during stress: effect of betablockade. Psychoneuroendocrinology. 2006 1 31;31(1):137–41. doi: 10.1016/j.psyneuen.2005.05.012 16046076 10.1016/j.psyneuen.2005.05.012

[51] Farag NH, Moore WE, Lovallo WR, Mills PJ, Khandrika S, Eichner JE. Hypothalamic pituitary adrenal axis function: relative contributions of perceived stress and obesity in women. Journal of Women's Health. 2008 12 1;17(10):1647–55. doi: 10.1089/jwh.2008.0866 19049359 10.1089/jwh.2008.0866 PMC2945932

[52] Strauss RS. Childhood obesity and self-esteem. Pediatrics. 2000 1 1;105(1):e15-. 10617752 10.1542/peds.105.1.e15

[53] Dutton GR, Bodell LP, Smith AR, Joiner TE. Examination of the relationship between obesity and suicidal ideation. International journal of obesity. 2013 9 1;37(9):1282–6. doi: 10.1038/ijo.2012.224 23318723 10.1038/ijo.2012.224 PMC3934959

[54] Hayran O, Akan H, Özkan AD, Kocaoglu B. Fat phobia of university students: attitudes toward obesity. Journal of allied health. 2013 8 29;42(3):147–50A. 24013244

[55] Almela M, Hidalgo V, Villada C, van der Meij L, Espín L, Gómez-Amor J, Salvador A. Salivary alpha-amylase response to acute psychosocial stress: The impact of age. Biological psychology. 2011 7 31;87(3):421–9. doi: 10.1016/j.biopsycho.2011.05.008 21664412 10.1016/j.biopsycho.2011.05.008

[56] Rohleder N, Wolf JM, Maldonado EF, Kirschbaum C. The psychosocial stress-induced increase in salivary alpha-amylase is independent of saliva flow rate. Psychophysiology. 2006 11 1;43(6):645–52. doi: 10.1111/j.1469-8986.2006.00457.x 17076822 10.1111/j.1469-8986.2006.00457.x

[57] Graves RE, Cassisi JE, Penn DL. Psychophysiological evaluation of stigma towards schizophrenia. Schizophrenia research. 2005 7 15;76(2):317–27. 15949664 10.1016/j.schres.2005.02.003

[58] Carroll D, Phillips AC, Lovallo WR. The behavioural and health corollaries of blunted physiological reactions to acute psychological stress: Revising the reactivity hypothesis Motivation Perspectives of Cardiovascular Response. APA Press, Washington, DC 2011:243–63.

[59] Olson MA, Fazio RH. Relations between implicit measures of prejudice: What are we measuring?. Psychological Science. 2003 11;14(6):636–9. doi: 10.1046/j.0956-7976.2003.psci_1477.x 14629698 10.1046/j.0956-7976.2003.psci_1477.x

[60] Crocker J, Major B. The self-protective properties of stigma: Evolution of a modern classic. Psychological Inquiry. 2003 10 1: 14(3–4):232–7.

[61] Sira N, Ballard SM. Gender differences in body Satisfaction: An examination of familial and individual level variables. Family Science Review. 2011;16(1):57–73.

[62] Bach DR, Flandin G, Friston KJ, Dolan RJ. Modelling event-related skin conductance responses. International Journal of Psychophysiology. 2010 3 31;75(3):349–56. doi: 10.1016/j.ijpsycho.2010.01.005 20093150 10.1016/j.ijpsycho.2010.01.005 PMC2877881

[63] Davison KK, Birch LL. Processes linking weight status and self-concept among girls from ages 5 to 7 years. Developmental psychology. 2002 9;38(5):735 doi: 10.1037//0012-1649.38.5.735 12220051 10.1037//0012-1649.38.5.735 PMC2530914

[64] White SE, Brown NJ, Ginsburg SL. Diversity of body types in network television programming: A content analysis. Communication Research Reports. 1999 9 1;16(4):386–92.

